# Thickness-dependent fast wetting transitions due to the atomic layer deposition of zinc oxide on a micro-pillared surface†

**DOI:** 10.1039/c9ra08498c

**Published:** 2020-01-06

**Authors:** Libing Duan, Xiangyang Ji, Yajie Yang, Sihang Yang, Xinjun Lv, Yanbo Xie

**Affiliations:** MOE Key Laboratory of Material Physics and Chemistry Under Extraordinary Conditions, School of Science, Northwestern Polytechnical University Xi'an 710072 China lbduan@nwpu.edu.cn ybxie@nwpu.edu.cn

## Abstract

Smart surfaces promote the fundamental understanding of wetting and are widely used in practical applications for energy and water collection. Light-induced switchable wettability facilitated by ZnO coatings, for instance, was developed for liquid manipulation at the surface. However, the transition of wetting states was reported to follow a hydrophobic–hydrophilic cycle in an hour, which is very long and may limit its future applications. We recently discovered that the cycle of the wetting state transitions on inorganic coatings can be shortened to less than 100 seconds by using ALD-coated ZnO on a pillared surface. However, the mechanisms are still unclear. Here, we investigated the effects of coating thickness on the transition speed and found that it significantly depended on the thickness of the coating with the optimal thickness less than 50 nm. We found that the minimum critical time for a wetting state transition cycle was less than 50 seconds with a thickness of 40 nm. Although the transition time of surfaces with coatings over 70 nm thickness remained constant at 10 min for a cycle, it was shorter than those of other deposition techniques for a coarse surface. Here, we propose a “penetration–diffusion” model to explain the fast and thickness-dependent wetting transitions. Our study may provide a new paradigm for fast wetting transition surfaces with cycle time within tens of seconds using a homogeneous thin layer coated on a rough surface.

## Abstract 2

Smart surfaces promote the fundamental understanding of wetting and are widely used in practical applications for energy and water collection.

## Graphical abstract

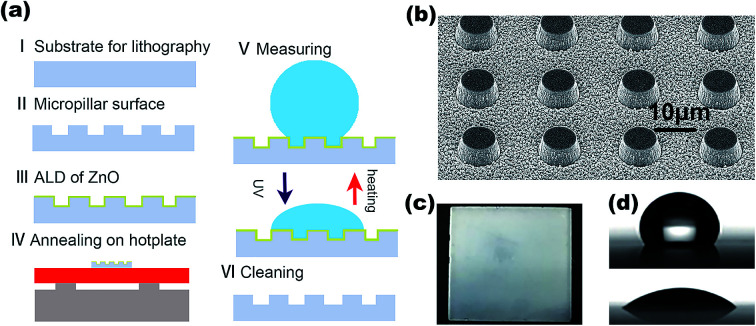

## Introduction

Wetting is a fundamental phenomenon, which has been studied for many decades.^1^ Inspired by the features of natural materials, many new materials and technologies have been invented to enable a smart-control of wetting states using artificial structures.^2–4^ The transition of materials between hydrophilic and hydrophobic states has attracted attention for its wide applications, such as self-cleaning,^5^ anti-fog coating,^6^ liquid transport^7^ and microfluidics.^8,9^ An external stimulus induces a change in the surface energy of materials, resulting in wettability transitions. The stimulus can originate from light, temperature, electrical fields, pH, magnetic field, *etc.*^10^ Light is an elegant way to regulate the wettability of materials due to its high precision,^11^ non-contact controllability and capability to create partially hydrophilic regions.^12^ Many photo-responsive materials, such as ZnO, TiO_2_, SnO_2_ and organic monolayers, have been used for manipulating the surface wettability transitions.^13–17^

Although it has been reported that the wetting transitions on an organic monolayer-coated surface like azobenzene can occur within tens of seconds, it is rarely reported in the case of inorganic coatings. We recently fabricated a surface with fast wetting transitions by depositing an atomic layer of ZnO on a micro-pillared surface.^18^ The hydrophobic to hydrophilic transition was completed within a minimum of 60 s, and the recovery on the hot plate was reduced to a minimum of 30 s. Our technique could also achieve a wetting transition speed for an inorganic coating, which was significantly higher than the results for those prepared by classic techniques, such as spray drying,^15^ vapor deposition,^19^ magnetron sputtering,^20^ sol–gel method^21^ and atomic layer deposition.^22^ However, the mechanisms underlying the fast wetting transitions are still unknown. In this work, we investigated the speed of wetting transitions as a function of the thickness of the ZnO layers deposited by ALD at various heating temperatures. We found the time of wetting transition was thickness-dependent and the optimal thickness for fast wetting transitions was between 10 to 70 nm. The transition speed on surfaces with thickness over 70 nm remained in the order of ten minutes, which is shorter than those obtained with the traditional coating techniques. Finally, we have theoretically illustrated the possible mechanisms of fast wetting transitions using a “penetration–diffusion” model, which relies on the penetration depth of the light and the diffusion length of the ions in the coating layer. Understanding the fast wetting transition mechanism may introduce a class of fast wetting transition surfaces formed from micro-pillared surfaces with nano-thick coating layers, which may be useful for liquid manipulations and the fabrication of smart surfaces.

## Fabrication and setup

**Fig. 1:**
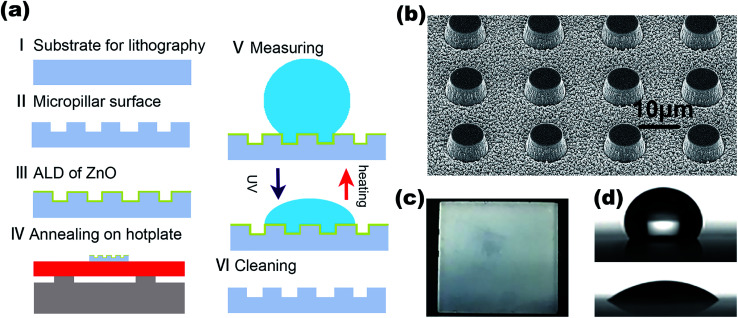
(a) Fabrication and CA characterization flow chart (including annealing). (b) SEM image of the micro-pillared surface. (c) The macroscopic image of the micro-pillared chip. (d) The typical contact angles at the hydrophilic (CA ∼ 120°, top) and hydrophobic states (CA ∼ 40°, bottom).

The fabrication process of the micro-pillared chips (Pyrex glass, 2 cm × 2 cm × 0.55 mm) is illustrated in Fig. 1a, showing individual microstructure fabrication and film coating steps. The micro-pillars were first etched by RIE (Plasma Pro 100 Cobra300) by standard photolithography and then coated with a ZnO film using an atomic layer deposition (T-ALD100A) system. The micro-pillars were characterized under a scanning electron microscope (FEI, Verios G4). The free-standing micro-pillar arrays with a center distance of 20 μm and 4 μm depth were fabricated, as shown in Fig. 1b. The substrates were carefully cleaned before each coating process to ensure that it is free of contaminants by following a standard process, which included immersion in dilute hydrochloric acid (20% purity) for 15 minutes, ultrasonic cleaning for 10 minutes, and finally, a cleaning cycle with acetone, alcohol, and deionized water in sequence.

The residual water droplets on the surface were dried using an N_2_ gun and sealed for ALD subsequently. By setting the number of coating cycles (∼0.16 nm per cycle) in the atomic layer deposition system program (diethyl zinc and DI water acted as precursors to provide zinc and oxygen, respectively), the film thickness could be precisely controlled. The coated chip is shown in Fig. 1c. After that, the contact angle reached the maximum with fast annealing in the air on a hot plate at 120 °C for 45 min. The thickness was optically characterized by visible light reflectometry (A3-SR-100, Apris technologies). For contact angle (CA) measurements of ZnO specimens with different thicknesses, we removed the existing ZnO coatings by repeating the cleaning process.

The wettability of the chips was evaluated from the CA values observed with a drop of 5 μL using a contact angle analyzer (POWEREACH, JC2000D2). A typical snapshot from the camera is shown in Fig. 1d to demonstrate the hydrophilic (top) and hydrophobic (bottom) states of the surface of our chips. To investigate the wetting transition speed, we characterized the contact angle after UV exposure or heating at specific time steps. To prevent film damage due to overheating, we performed the experiments in a sequence of temperatures from low to high. UV irradiation was achieved using an uncollimated ultraviolet light source (145 mW cm^−2^, EPILEDS) placed at 3 cm from the chips in ambient temperature.

## Results

**Fig. 2:**
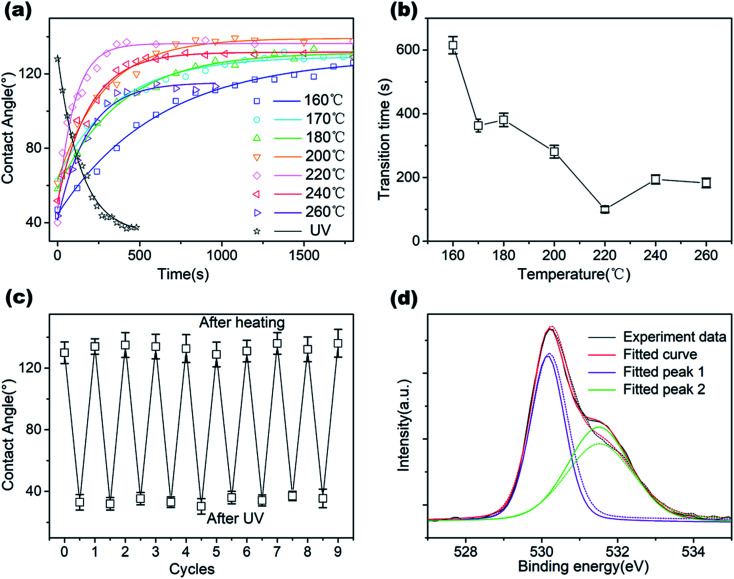
(a) Wetting transition kinetics with 70 nm ALD-coated ZnO under heating (color dots) and UV irradiation (black dots), showing exponential fitting (solid lines). (b) Critical Transition Time (CTT) changes as a function of temperature. (c) Reversible wetting-state transitions under UV irradiation or heating. (d) XPS characterizations indicates a clear change in the peak of O 1s before (dashed line) and after (solid line) UV irradiation for 2 min.

We considered the wettability transitions of the 70 nm-thick ZnO coated micro-pillared chips as an example. The chips were first placed under UV irradiation (145 mW cm^−2^ with a center wavelength of 365 nm) to investigate the transition from (initial) hydrophobic state to hydrophilic state, shown as black dots in Fig. 2a. CA was measured immediately after UV exposure, and the residual water was blown before subsequent UV irradiation. The time steps indicate the overall duration of exposure under the UV lamp. We found that the exponential decay function fitted well with the CA values measured as a function of time: 
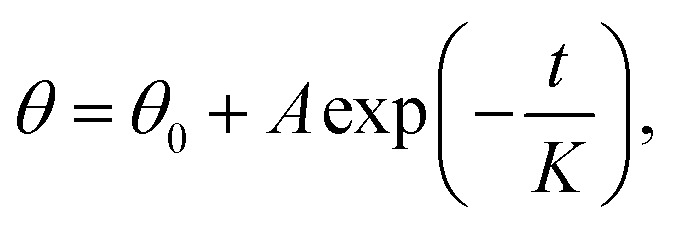
 where *θ*_0_, *A*, *K* are the saturated value at the hydrophilic state, the constant value obtained by fitting, the critical transition time to attain saturation, which represents the speed of transition. A similar tendency of exponential decrease of CA has been reported for TiO_2_ sol–gel coatings using a comparable statistical analysis to demonstrate the critical transition time (CTT); however, with the cycle of wetting transition extending to an hour.^23^ As shown in Fig. 2a, we could derive the CTT from a hydrophobic to hydrophilic state as 156 seconds, which is longer than that observed previously for 40 nm thick coatings (60–80 seconds).^18^ The measured data with 40 nm thickness of the coating was from our previous work for comparison with the critical wetting transition time between different thicknesses. We suspected that it may be relevant to the thickness of the ZnO coating, as discussed below.

We further investigated the recovery process from 160 °C to 260 °C by placing the chips on a programmable hotplate for thermal treatment. We divided the total time of the chips on the hot plate into time step (60–120 s) depending on the speed of transitions and investigated the change in CA during the recovery process. CA measurements were done after cooling the chips to room temperature. As we demonstrated previously, the temperature of the chips reached equilibrium within 0.5 seconds after placing on the hotplate, which could be ignored while considering the time of heating.^18^ We found that the contact angle, after the thermal treatment, increased rapidly at the beginning and then, tended to reach a stable value, matching well with the exponential decrease function: 
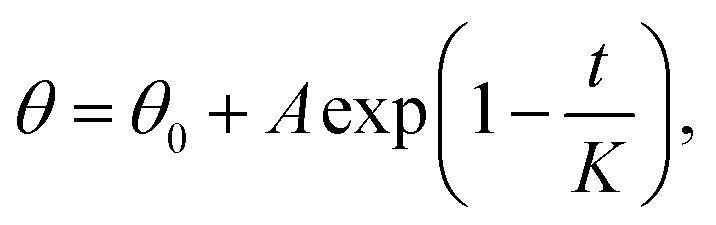
 where *θ*_0_, *A*, *K* are the saturated value at hydrophobic state, the constant value obtained by fitting, and the CTT value for hydrophilic to hydrophobic transition. As the temperature increased, the speed of transition gradually became faster. When the temperature increased to 260 °C, the wetting transitions were irreversible with a lower maximum CA after heating for 960 s, which possibly due to the damage caused by overheating. The ZnO coating on the surface no longer covered the pillars homogeneously, thereby exposing the glass substrate and showing a decrease in the maximum contact angle of the substrates. From the CTT values in Fig. 2a, we could find that the transition speed of wettability is a function of heating temperature in Fig. 2b. With an increase in the temperature, CTT gradually decreased from the initial 600 s to 100–200 s and remained saturated. The surface demonstrated excellent repeatability of the wetting state transitions for over 9 cycles (Fig. 2c), with the contact angles ranging from 30° to 130°, which demonstrated that the surface is promising for future applications. To investigate the mechanisms of the fast transitions, we characterized the morphology changes of the 70 nm-thick ZnO coating on a flat glass slide by atomic force microscopy. The results from the averaged surface roughness did not show a clear change due to UV exposure and the annealing process (ESI S1†).

Then, XPS was used to investigate the changes in the chemical properties of the surface before and after UV irradiation. A clear change in the shoulder peak at 531.5 eV appeared in the results (Fig. 2d), which indicated that UV irradiation generated oxygen vacancy and absorbed water.^24^ Although we demonstrated successful wetting transitions on the 70 nm-thick ZnO-coated pillared surface, the response time of the wetting transition was slower than previously reported results, which inspired us to investigate the effect of film thickness on CTT.

**Fig. 3:**
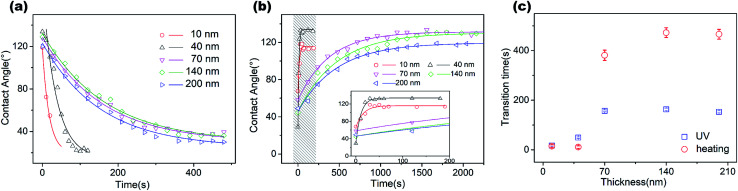
(a) Hydrophobic–hydrophilic transition kinetics with different thickness of coating and change in irradiation time. (b) Hydrophilic–hydrophobic transition kinetics with thickness change from 10 nm to 140 nm under heating at 180 °C, showing exponential fitting (solid lines). The transition kinetics of 10 nm and 40 nm coatings are presented in the inset figure. (c) Fitted data from (a) and (b) showing that the critical transition time increases with the thickness and reaches saturation.

For this, we prepared ZnO coatings on the same substrate with layer thicknesses of 5 nm, 10 nm, 40 nm, 70 nm, 140 nm and 200 nm and found successful reversible transitions in all except the 5 nm-thick coating. To avoid contaminations during measurement, we removed the ZnO coating and carefully cleaned the substrate before coating a ZnO layer of different thickness. We repeatedly UV irradiation to measure the speed of transition from hydrophobic to a hydrophilic state by using time steps (Fig. 3a), while the power density remained at 145 mW cm^−2^ for all thicknesses of the coating.

The initial contact angle for each sample dramatically decreased from 120° to ∼30° under UV irradiation, exhibiting the law of exponential decay. The fast decrease at the beginning of the UV process was attributed to the high-speed yielding of electron–hole pairs on the initial surface.^16^ Our measurements presented a clear decrease in transition speed with the thickness increasing from 10 to 70 nm, but reached a plateau for thicknesses over 70 nm. We fitted the exponential decay function for each set of experimental results. The fitted results are presented in Fig. 3c (blue dots). It should be noted that the unstable wetting state at the thickness of 5 nm was possibly due to the inhomogeneity of the film, which didn't cover the surface of the substrate completely. A similar phenomenon was observed by Ahn *et al.*, who reported that the initial growth stage of the film deposited by the ALD method showed island-like spots due to the random adsorption of reactants on the substrate.^25^ The film became homogeneous when the thickness was over 7.5 nm, showing a uniform layer growth mode and fully-covered coating.

We then performed heat treatment by placing the chips on a 180 °C hot plate to achieve hydrophilic to hydrophobic state transition and derived the effects of film thickness on the transition speed (Fig. 3b). To clearly show the fast transition for the thin layer of the coating, an amplified view of the 10 nm- and 40 nm-thick coating samples are shown in the inset of Fig. 3b. The speed of transition obviously changed with the thickness, with the excellent recovery of CA at the hydrophobic state. It showed that the chips with a thin layer (10–40 nm) of the coating were able to complete a cycle of wetting state transition process within 46 s, which is apparently shorter than the specimens with coating thicknesses over 70 nm (6–7 min). The small difference in the transition speed between the 10 nm and 40 nm coatings is possibly caused because the time of experimental operation is comparable to the wetting transition time (ten seconds).

From the CTT of both UV and thermal transition processes, we could clearly demonstrate the transition speed as a function of thickness, as shown in Fig. 3c. We found the same tendency for both the transition processes with changes in thickness. The CTT dramatically increased with film thickness and then, reached a saturated value. For a complete transition cycle of the hydrophobic and hydrophilic states, it took a minimum of 46 seconds to 10 min as the thickness increased from 10 to 70 nm, and saturation was attained when the thickness was over 70 nm thick (Fig. 3c). There are a few things need to be noted. The CTT of both hydrophilic to hydrophobic and reverse transitions had the same tendency as the function of film thickness. Besides, the longest transition cycle (∼10 min) is still obviously shorter than those observed for coarse surfaces obtained with the other deposition techniques, especially from hydrophilic to hydrophobic state transition by the heating process.^26,27^ The mechanism of fast transition is critical for further fabrication of fast wetting transition surfaces and relevant applications, which is discussed in the following section with a possible explanation for the thickness-dependent change in wetting transition speed.

## Discussion

As described above, the transition of wettability relies on the surface energy changes caused by UV excitation or the annihilation of vacancies on heating. We attributed the fast transition in a wide range of CA to the thin coating of ZnO on the rough surface of micro-pillars machined on a glass substrate. The wetting states in our designed pillars can be categorized to be in the Wenzel state, which was optically observed and matched to the classical wetting theories.^18,28^ Young's equation illustrates the effects of roughness, showing that the apparent contact angle θ* is relevant to the roughness ratio (*r*) and Young's contact angle (*θ*) of an ideal surface, as shown below.^29^1
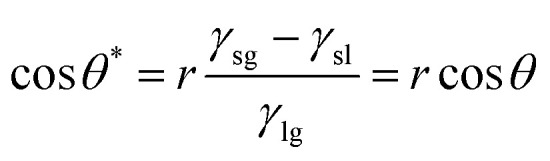
where *γ*_sg_, *γ*_sl_ and *γ*_lg_ are the interfacial tension of the solid–gas, solid–liquid, and liquid–gas interfaces, respectively. The micro-pillared surface (*r* > 1)^30^ with ZnO coating deposited by the ALD method can be well explained according to Young's equation, amplifying the range of the contact angle transition.

In this section, we discuss the possible mechanisms of the thickness-dependent fast wetting transition and comparison with other film deposition techniques (*e.g.* sol–gel) by a “penetration–diffusion” model. Two critical physical lengths need to be noted. It has been reported that the UV light can only penetrate *δ* (typically less than 60 nm)-thick ZnO,^31^ inducing photon-excited vacancies within this penetrated layer. The vacancies diffuse to an average length *L*_p_ over their lifetime,^32^ which can be estimated by *L*_P_ = *D*_P_*τ* ∼ 50 nm (*μ*_p_ = 1 cm^2^ V^−1^ s^−1^, *τ* = 1 ns)^33^ and is comparable to the penetration distance. Since the surface energy is determined by the concentration of vacancies and the quantity of absorbed water, the generation and diffusion of vacancies, which are thickness-dependent, are directly relevant to wettability.^34^

### Hydrophobic–hydrophilic transition

We first discuss the wetting transitions from hydrophobic to a hydrophilic state under UV exposure. To clearly illustrate the thickness-dependent wetting transitions, we classified the samples into two models – thin film and thick film models. The thin film model was defined to have less thickness than the critical thickness of the penetration depth of the UV light (wavelength of 365 nm), while the thick film was considered to have a film thicker than the sum of penetration depth and diffusion length (>100 nm), where the coated thickness in-between is the transition state from thin film model to thick film model.

#### Thin film model

**Fig. 4:**
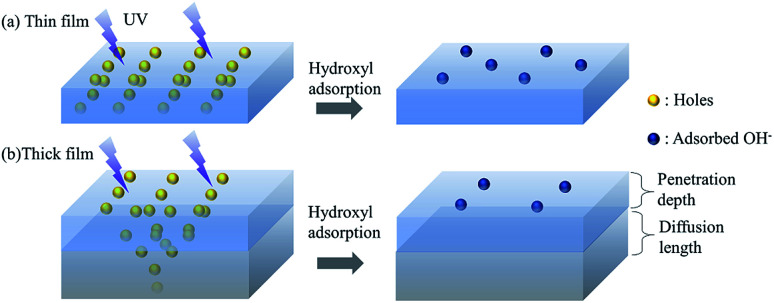
A schematic diagram of the penetration–diffusion models for the generation of vacancies.

In the thin film model, since the photons can penetrate the entire ZnO film to generate vacancies throughout the depth of the film (Fig. 4a), the diffusion of generated vacancies can be ignored for the change in CA. As the change in wettability is determined by the generated concentration of vacancies, CTT is determined by the light power density and thickness. Here, we used the scaling analysis to estimate the time of transition.^35^ The total irradiation energy *E* absorbed by a thin film to reach saturation can be qualitatively described as:

2 *E* ∝ *CPtS*

where *P*, *t*, *S*, and *C* are the UV power density, CTT, exposed area and a constant relevant to the absorption coefficient, respectively. Incorporating the area formula (*S* = *V*/*h*) into the above equation, we can derive the dose of absorbed UV light (absorbed energy per unit mass)^36^ to achieve saturation, which is dependent on the total mass of the thin film shown, as given below:3
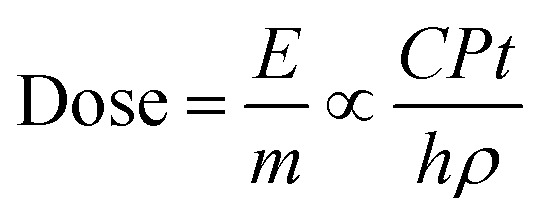
where *V*, *h*, *ρ*, and *m* are the volume, thickness, density, mass of the film, and C is a constant value independent of UV irradiation. For a homogeneous coating, the dose for reaching a saturation equilibrium is dependent on the total mass of the thin film, dependent on the intrinsic properties of the coatings.^37^ Hence, it can be considered that the saturation time is qualitatively proportional to the thickness under a constant light power density. The high illumination power and thin film help in increasing the saturation time of the vacancies. Hence, in the thin film model, the transition of wetting states can be faster than that in the thick film.

#### Thick film model

However, for the thick film model, we need to consider the diffusion of the vacancies. Since the vacancies can only be generated within the penetration depth from the surface (Fig. 4b), the vacancies can diffuse into the inner layer of ZnO due to the gradient from the surface until annihilation. Thus, the concentration of vacancies at the surface decreases, reducing the surface energy and the number of absorbed hydroxyl groups,^38^ eventually slowing down the speed of wettability change. To reach a saturated state, the thick film model requires a higher dose of UV irradiation, considering the diffusion of vacancies.

To quantitively discuss this transition in the thick film model, we used the vacancy distribution under one-dimensional stable diffusion, which can be described as:^39^4
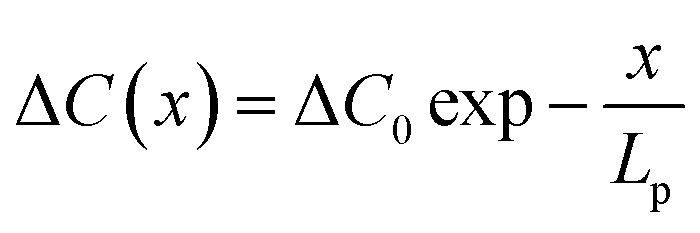
where Δ*C*(*x*) is the concentration at a distance *x* from the surface, which is also a critical parameter in the photocatalytic process.^40^Eqn (4) indicates that the distribution of vacancy density exponentially decreases due to diffusion and hence needs a higher dose and time of stimulation to reach an equilibrium state for CA transitions.

According to the thick film model, the wettability transition is determined by the sum of penetration depth and diffusion length. Thus, the generation of vacancies and the density of hydroxyl adsorption are independent of the film thickness when the thickness increases further. The penetration–diffusion model can possibly also explain the hydrophobic to hydrophilic transition process for classic deposition techniques (like sol–gel), which have a similar transition speed to the thick film model of the ALD-coated ZnO surface on the micro-pillared surface (within a few minutes),^41,42^ as the saturation time of generated vacancies are determined by the penetration–diffusion length at the surface irrespective of the thickness of the ZnO film.

### Hydrophilic–hydrophobic transition

#### ALD coating

The recovery process from a hydrophilic to a hydrophobic state can be explained by the thin and thick film models. As described above, as the temperature increases, the surface evolves to the original state, reducing the surface energy and increasing the contact angle. Since the transition speed of CA depends on the annihilation process of the vacancies, the thickness is critical to the transition speed. In a thin film, the vacancies are annihilated faster in a thinner film^43^ since the diffusion of vacancies in the “bulk” can be ignored. This matches the experimental observation of CA transitions when the film thickness is less than 40 nm. However, the vacancies only exist in the penetration and diffusion depth and hence become independent of the thickness, which is observed from the results of thick coatings over 70 nm. High temperatures can accelerate the speed of annihilation and can also possibly damage the homogeneity of the coatings, thus inducing irreversibility of wetting states.^44^

#### Coarse surface

It should be noted that the maximal CTT for the ALD-coated ZnO surface was less than 10 min, which is apparently shorter than the transitions in the samples with a course surface prepared by other techniques (typically in the order of an hour), such as sol–gel and other physical depositions.^45,46^ Here, we discuss the mechanism of fast transition in the ALD-coated samples.

It is well-known that the existence of nanostructures, such as nano-needles or nanoparticles, on a rough surface enhances the range of wettability transitions, according to the wetting theories. However, there are side effects of the wetting transitions on these deposited coarse surfaces with nanostructures. It has been reported that the conduction and migration of vacancies in the nano-confined areas show slower carrier mobility than that in the bulk.^47,48^ This may limit the speed of diffusion and annihilation of vacancies, inducing a longer time for equilibrium and CA transition on the surface. However, the ALD coating has a homogeneous surface and is in close contact with the substrate, where the micro-pillars perform the role of a rough surface. The film has good contacts with the substrate, avoiding the diffusion of ions in the nano-confined space, resulting in a faster transition for the recovery process. In addition, for the recovery process, the low thermal conduction of the nano-needles seems to be another possible reason,^49^ slowing down the diffusion and annihilation of vacancies in the nano-needles. For coarse depositions, these nanostructures enhance the range of wettability change. On the contrary, we suspect that due to the joint action of slow migration and poor thermal conduction at the nanostructures, the wetting transition in a rough coating is slower than the homogeneous coating achieved by ALD on a micro-pillared surface. The latter technique possibly can overcome these side effects for the optimization of a fast superhydrophobic–superhydrophilic wetting transition and liquid manipulations in the future.

## Conclusion

In this paper, we investigated the wettability transitions of ALD-coated ZnO film on a micro-pillared surface. We found the transition speed was thickness dependent, with a minimum of 35 seconds for hydrophobic to hydrophilic transition under UV irradiation and 11 seconds for recovery from the hydrophilic to hydrophobic state at 40 nm coating thickness. As the film thickness increased, the transition speed remained saturated at 10 min per cycle and was independent of the thickness. The transitions of the ALD-coated micro-pillared surface were much faster than those of other types of deposition techniques. We propose a penetration–diffusion model to explain the fast wetting transition process, which can explain the thickness-dependent wetting transition of the ALD coatings and the difference between ALD coated sample and those coated using other techniques. Our discovery can possibly provide a new paradigm for fast wetting transition surfaces for the manipulation of liquids and the development of smart surfaces due to its fast response, excellent repeatability and ease of fabrication.

## Conflicts of interest

The authors declare no competing financial interest.

## Supplementary Material

RA-010-C9RA08498C-s001
