# Supplementary material for: Thickness-dependent fast wetting transitions due to the atomic layer deposition of zinc oxide on a micro-pillared surface
Source: RSC Adv. 2020 Jan 6;10(2):1120–6. doi: 10.1039/c9ra08498c (PMC9048290; doi:10.1039/c9ra08498c)
Supplement: RA-010-C9RA08498C-s001 [file RA-010-C9RA08498C-s001.pdf]

Electronic Supplementary Material (ESI) for RSC Advances.
This journal is © The Royal Society of Chemistry 2019

Thick-dependent fast wetting transitions by atomic layer deposition of zinc oxide at micro-pillared surface

Libing Duan,*[⊥a] Xiangyang Ji,[⊥a] Yajie Yang,[a] Sihang Yang,[a] Xinjun Lv,[a] and Yanbo Xie[a]*

a. MOE Key Laboratory of Material Physics and Chemistry under Extraordinary Conditions, School of Science, Northwestern Polytechnical University, Xi'an 710072, China;

Supporting information

S1. The morphology changes of 70nm ZnO of ALD on a flat glass slide by Atomic Force Microscope

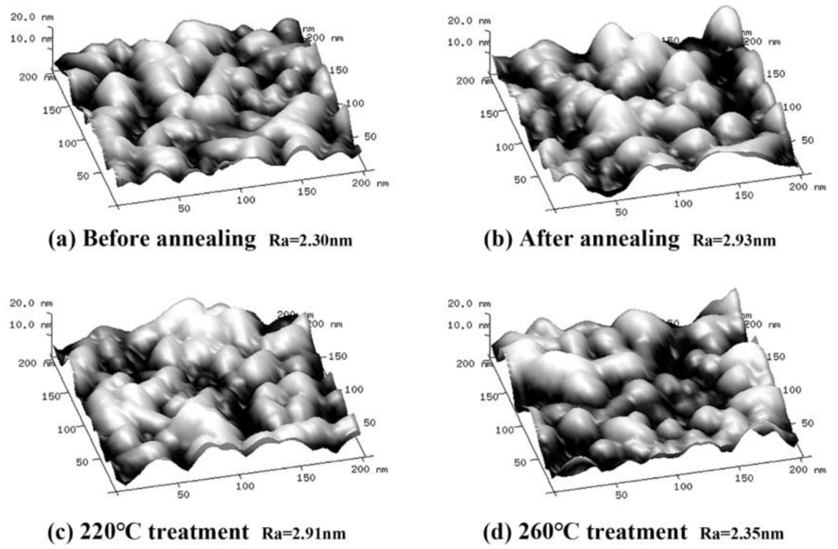

Fig S1. The morphology after various heating condition. (a)Before annealing. (b)Annealing at 120°Cfor 45min.(c) Heating at 220°C for 30min after annealing. (d)Heating at 260°C for 30min after annealing.

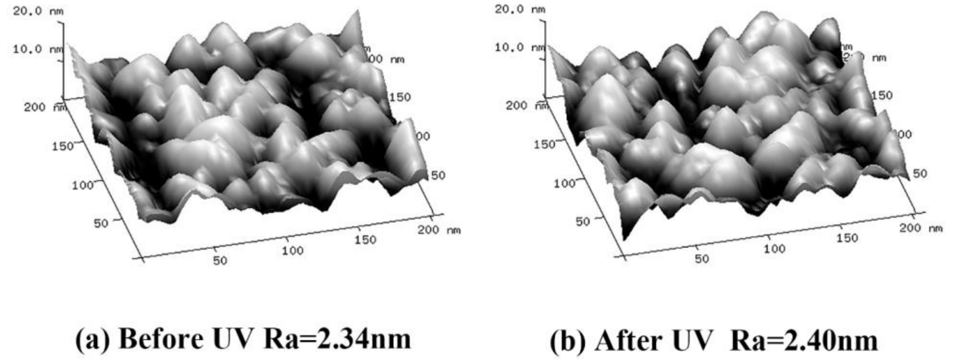

Fig S2. Effect of UV on morphology. (a)Before UV exposure. (b) After UV exposure for 2min.

The average surface roughness of samples didn't show any changes under UV exposure and annealing process.
